# Gambling on others’ health: risky pro-social decision-making in the era of COVID-19

**DOI:** 10.3389/fpsyg.2024.1370778

**Published:** 2024-09-19

**Authors:** Leyla Loued-Khenissi, Corrado Corradi-Dell’Acqua

**Affiliations:** ^1^Theory of Pain Laboratory, Faculty of Psychology and Educational Sciences, University of Geneva, Geneva, Switzerland; ^2^Department of Clinical Neuroscience, University Hospital of Lausanne, Lausanne, Switzerland; ^3^Center for Mind/Brain Sciences, University of Trento, Rovereto, Italy

**Keywords:** risk, decision-making, other-regarding behavior, COVID-19 pandemic, uncertainty

## Abstract

**Introduction:**

In the early days of the COVID-19 pandemic, individuals were asked to perform costly actions to reduce harm to strangers, even while the general population, including authorities and experts, grappled with the uncertainty surrounding thenovel virus. Many studies have examined health decision-making by experts, but the study of lay, non-expert, individual decision-making on a stranger’s health has been left to the wayside, as ordinary citizens are usually not tasked with such decisions.

**Methods:**

We sought to capture a snapshot of this specific choice behavior by administering two surveys to the general population in the spring of 2020, when much of the global community was subject to COVID-19-related restrictions, as well as uncertainty surrounding the virus. We presented study participants with fictitious diseases varying in severity that threatened oneself, a loved one or a stranger. Participants were asked to choose between treatment options that could either provide a sure, but mild improvement (sure option) or cure the affected person at a given probability of success (risky option).

**Results:**

Respondents preferred gambles overall, but risk-seeking decreased progressively with higher expected severity of disease. This pattern was observed regardless of the recipient’s identity. Distinctions between targets emerged however whendecisions were conditioned on a treatment’s monetary cost, with participants preferring cheaper options for strangers.

**Discussion:**

Overall, these findings provide a descriptive model of individual decision-making under risk for others; and inform on the limits of what can be asked of an individual in service to a stranger.

## 1 Introduction

In December 2019, an unprecedented outbreak of pneumonia caused by the SARS-CoV-2 virus, emerged in the city of Wuhan (China). This disease, known as COVID-19, rapidly spread throughout the globe, finding authorities, healthcare providers and lay individuals woefully unprepared, leading to a high degree of uncertainty (Lorettu et al., 2021). By March 2020, many countries had put restrictive measures in place such as lockdowns, curfews, social distancing and quarantines to contain the virus’ spread. These measures had economic and psychological consequences on the people involved (Kunzler et al., 2021; Lima et al., 2020; Nochaiwong et al., 2021; Santomauro et al., 2021; Wu et al., 2021), with some individuals more affected than others (Adams-Prassl et al., 2020; Blundell et al., 2020; Daly et al., 2020). At the same time, the effect of the virus was not uniformly distributed, changing as a function of age and pre-existing medical conditions (Du et al., 2020; Yancy, 2020). As such, the early days of the pandemic presented a stark dilemma to lay-people: how to act for the sake of another in the face of uncertainty, and at what cost? This problem spilled over into socio-political discourse (Barbieri and Bonini, 2020; McKee and Stuckler, 2020; Wolff, 2022) and even prompted acts of violence (Choi and Lee, 2021; Elfrink, 2020; Taylor and Asmundson, 2021). But the pandemic also offered a real-time opportunity to assess costly, individual, pro-social, risky decision-making in instances where even authorities were subject to uncertainty.

### 1.1 Economic decision-making under risk

Uncertainty has been extensively studied in economic decision-making (Johnson and Busemeyer, 2010; Loued-Khenissi and Preuschoff, 2020; Preuschoff et al., 2013) commonly in the form of “risk,” an expected uncertainty based on event probability. Within this framework, an event is deemed riskier the more unpredictable it is. Hence, high risk differs from predictable danger, which refers to conditions where individuals can easily foresee what will occur. Theoretical and empirical accounts within the domain of economic decision-making (Allais, 1953; Bernoulli, 1738/1954; Cox and Sadiraj, 2008; Tversky and Kahneman, 1992) show that risk steers decision-making away from maximizing predicted gains: agents are risk-averse in the gain domain, showing a preference for sure options, while they are risk-seeking when facing loss. Importantly, risk-seeking decreases linearly with expected monetary loss, a robust phenomenon that has been replicated across different countries (Huck and Müller, 2012; Ruggeri et al., 2020). It remains unclear whether such risk preferences are conserved when deciding for others.

Several empirical studies (Andreoni and Miller, 2002; Cutler and Campbell-Meiklejohn, 2019; Rand and Nowak, 2013) as well as human social structure (Tomasello et al., 2012), provide evidence that other-oriented decisions often violate the *homo economicus* model (Samuelson, 1993). Individuals commonly engage in costly actions to cooperate with others (Diekmann, 2004; Fehr and Fischbacher, 2004) or to help people in need (Soetevent, 2005), albeit by investing less resources than those usually mobilized for one’s own benefit (Lockwood et al., 2017). Furthermore, several studies have found that decisions made on behalf of others resemble those made for the self (Civai et al., 2010, 2012; Corradi-Dell’Acqua et al., 2013, 2016), thus suggesting an individual ability to put oneself in the shoes of unknown others. When looking specifically at risk preferences, a recent meta-analysis reveals a strong variability in the effects described in the literature, all converging toward an overall trend of slightly enhanced risk-seeking for others relative to one’s self (Polman and Wu, 2020).

### 1.2 Health decision-making under risk

The role of uncertainty in decision-making also extends to health choices (Reis and Spencer, 2019). In this context, experts, such as authorities or physicians specifically trained in assessing risk, are usually those tasked with making decisions. For instance, authorities may rely on the Precautionary Principle, assuming a risk-averse stance toward public health (Goldstein, 2001; Gollier and Treich, 2003; Sunstein, 2005). Other health evaluations commonly use expected utility (Abellan-Perpiñan et al., 2009; Evans and Viscusi, 1991; Levy and Nir, 2012; Meltzer, 2001; Russell and Schwartz, 2012). For example, the Quality-Adjusted Life-Years (QALY) is a form of expected utility integrating time that guides cost analysis (Bleichrodt, 1997) and health policy implementations (Mooney, 1989; Pinto-Prades et al., 2019). With both the Precautionary Principle and QALYs, the calculus employed in the service of making a decision is explicit. However, the novelty and virulence of Sars-Cov-2 imposed the burden of costly decision-making on people’s health under uncertainty on lay people, leaving the question open as to what strategy is used in such a context. Tversky and Kahneman (1981) speculated that individuals would be risk-seeking for treatment when facing an infectious disease, mirroring choice behavior when facing monetary loss. However, whereas some studies investigating pain-management choices confirm this prediction (Loued-Khenissi et al., 2022), others show that individuals are risk-averse for their own treatment options (Hellinger, 1989) or for choices that could influence their life expectancy (Attema et al., 2013). Finally, meta-analyses suggest that health decision-making in medical contexts lead to a shift toward more cautious (risk-averse) decisions for others relative to the self, in contrast to what is found in monetary/managerial scenarios (Atanasov, 2015; Polman and Wu, 2020). However, the medical contexts included in these meta-analyses involved primarily physicians (or people acting as physicians) choosing for patients (Atanasov, 2015; Polman and Wu, 2020), thus still focusing on decision-making processes in professionals, rather than the lay individual. To the best of our knowledge no studies have tested how ordinary people make risky decisions on disease treatments for unknown others.

### 1.3 The present research

Here, we investigated uncertainty’s role in costly decision-making for people’s health by applying an expected value model of disease severity in a probabilistic task. We administered two anonymous surveys to the general population between May and July, 2020, when at least half the global population was under confinement and grappling with questions on how to personally respond to the COVID-19 pandemic and how much to sacrifice in service of the greater good. We presented respondents with fictitious diseases and their associated risks of contraction, along with different, costly treatment options. Participants were asked to choose between a treatment that avoids contracting the disease at a given probability (risky option) or one that mitigates symptoms with 100% effectiveness (sure option). Building on a well-established literature from economic decision-making (Johnson and Busemeyer, 2010; Loued-Khenissi and Preuschoff, 2020; Preuschoff et al., 2013), we define a treatment as riskiest when outcome (negatively or positively valenced) probability approached 0.5. Respondents made decisions for themselves (*Self*), a loved one (*Beloved*), and a *Stranger*. In this design, the *Self* condition represents the baseline against which we compare choices made for others (either the *Beloved* or a *Stranger*). In particular, choices made for an unknown person (relative to one’s self) are of key interest, as they provide a snapshot on individual, costly, risky decisions for a stranger’s wellbeing. We included a loved one as a target to further characterize self-other differences, and to investigate effects related to the social proximity of the deciding agent. Following the literature reviewed above, we sought to test the following three hypotheses. First, we expect that, in the *Self* condition, individuals would display an overall risk-seeking stance that progressively declines with increasing expected disease severity (*Hypothesis 1*). This prediction is directly derived from studies arguing that choices on disease contraction mirror those observed for monetary losses (Tversky and Kahneman, 1981). Second, we predict that decisions for strangers would be more risk-averse than those associated with the self (*Hypothesis 2*). This is motivated by previous studies meta-analyses testing risk decision-making in medical contexts (Atanasov, 2015), and pain management (Loued-Khenissi et al., 2022). Third, and consistent with our prior research (Loued-Khenissi et al., 2022), we expect social proximity between self and other to influence the results, with agents acting for their loved ones as they would for themselves (*Hypothesis 3*).

## 2 Survey 1

### 2.1 Methods

#### 2.1.1 Population

The survey was made available online to individuals 18 years and older. Respondents were recruited through the Prolific.co platform.^1^ Participation was voluntary and compensated between £1.5 and £2 (i.e., £5/h on an average completion time of 22 min). The Ethics Committee of the Faculty of Psychology and Educational Sciences of the University of Geneva approved the study.

Survey 1 was an exploratory investigation to obtain a first snapshot of decision-making for others’ well-being. Within a week (in May 2020), 381 participants in the Prolific.co platform began the survey, and 366 completed the questionnaire. We excluded an additional 22 participants for making attentional errors (see data analysis below for more details) or providing implausible answers (e.g., listing a non-human as a loved one), leaving *n* = 344 for analysis [43% F; mean age 25 (IQR = 10)]. Cohort characteristics are described in Table 1.

**Table 1:** Surveys 1 and 2 cohort details.

	Survey 1	Survey 2	Comparison
Gender	43% females	64% females	χ^2^ = 20.45^***^
Participant’s age	25 (iqr = 10)	26 (iqr = 10)	*t* = −1.29
Beloved’s age	30 (iqr = 29)	32 (iqr = 28)	*t* = −0.49
# countries represented	42	44	χ^2^ = 0.06
Monthly income	$2,499 (iqr = $3250)	$3,499 (iqr = $6500)	*t* = −4.25^***^
Pandemic-related monetary loss	$200 (iqr = $1000)	$3.5 (iqr = $2000)	*t* = 0.99
Perceived adequacy of confinement measures *(most frequent response)*	Adequate (69.60%)	Adequate (63.75%)	χ^2^ = 1.67
Job-loss due to COVID-19	27	16	χ^2^ = 0.44
Positive to COVID-19	4	2	χ^2^ = 0

Continuous variables are described in terms of median and inter-quartile range (iqr). Group differences are estimated in terms of χ^2^ test (for proportions) or independent sample *t*-test (for continuous variables). Significant effects are highlighted in bold, with *** corresponding to *p* < 0.001.

#### 2.1.2 Procedure

As a first step, participants accessed an informed consent page. By selecting the option “I accept,” they were then directed to the main survey. This was an adaptation of standard lottery tasks from economic decision making (e.g., Tversky and Kahneman, 1992) to the context of a pandemic, where respondents had to choose between different treatments that could either dampen or cure disease at given effectiveness rates. We specifically chose scenarios involving treatments for fictitious diseases, so as to freely manipulate probabilities in a plausible fashion. This manipulation would not have been possible had we employed real diseases (which participants might have prior knowledge of), or non-pharmacological protective behavior like wearing masks or abiding to self-confinement (for which precise probabilities might have appeared implausible). The survey contained 45 items, each describing a risk of contracting a given disease. For each item, respondents chose one of 3 treatment options. Below is an example:

“*Your loved one has a 25% chance of contracting and falling severely ill with disease F. Symptoms include high fever, muscle pain and vomiting of blood. Standard treatment requires a two-week hospital stay in intensive care. The illness leaves minor but lasting cardiac deficits and a slight but permanent hearing impairment. You can:*

- *Do nothing and let your loved one face the initial chance of falling severely ill with disease F*
- *Pay half your monthly salary for additional treatment that will certainly reduce the severity of the illness, such that it leaves only a slight but permanent hearing impairment*
- *Pay one tenth of your monthly salary to halve the risk of contracting the illness altogether with an experimental treatment*.”

The scenarios described 5 possible diseases, with different risks of contraction (p_D_; in the example above, 0.25), and levels of severity (S_D_; either death or severe lasting deficits). Diseases were also described according to their symptoms, which were loosely based on real world infectious diseases (C for Chikingunya; D for Dengue; E for Ebola; F for Flu, etc.), with, however, fictitious morbidity and mortality rates. Each item threatened one of three possible targets (*Self, Beloved*, or *Stranger*), and were followed by 3 possible options:

- Do nothing and let the person face the initial risk of disease;
- Pay an amount of money for a known treatment that partially reduces disease severity (sure option);
- Pay an amount of money for an experimental treatment that reduces the risk of initial contraction (the gamble).

The gamble cost was always set to one tenth of the respondent’s monthly income. Sure treatment options for each disease varied in price, between 0.1, 0.5, or 1 unit of the respondent’s monthly income. Selecting the sure option treatment for diseases with a mortal risk (C, D, and E from Table 2) reduced prognosis to severe lasting deficits. Known treatment reduced prognosis to mild lasting deficits for diseases that carried severe lasting deficits (F and M) (see Table 2). Four attentional catch questions were interspersed across the survey to ensure respondent engagement (e.g., “Is 7 > 3?”). All items were randomized across diseases and targets.

**Table 2:** Disease items and treatment options.

	Disease	Prognosis (SD)	Contraction risk (pD)	Sure option prognosis	Gamble success
Survey 1	C	Death	5%	Minor, lingering cardiac deficits and slight, but permanent hearing impairment	75%
	D	Death	25%	Minor, lingering respiratory deficits and slight, but permanent visual impairment	75%
	E	Death	5%	Minor, lingering respiratory deficits and slight, but permanent visual impairment	50%
	F	Minor but lasting cardiac deficits, and a slight but permanent hearing impairment	25%	Slight but permanent hearing impairment	50%
	M	Minor but lasting respiratory deficits and a slight but permanent visual impairment	10%	Slight but permanent visual impairment	75%
Survey 2	C	Death	5%	Minor, lingering cardiac deficits and slight, but permanent hearing impairment	75%
	D	Death	25%	Minor, lingering respiratory deficits and slight, but permanent visual impairment	75%
	P	Minor but lasting cardiac deficits and a slight but permanent hearing impairment	50%	Slight but permanent hearing impairment	50%
	M	Minor but lasting respiratory deficits and a slight but permanent visual impairment	10%	Slight but permanent visual impairment	75%

We also collected participants’ non-identifying demographic information (country of residence, age and gender, and household monthly income and education), that could impact costly, pro-social decision-making (Boschini et al., 2018; Freund and Blanchard-Fields, 2014; Wiepking and Breeze, 2012). We also asked participants to identify a loved one (*Beloved*) by their role. The survey was designed using LimeWire software, and was fully anonymous; and available in English, French and Italian (English version available under the Open Science Framework https://osf.io/9fjdq/).

#### 2.1.3 Data analysis

Analyses were performed using R 4.1.2 freeware software;^2^ de-identified data and processing scripts are available at: https://osf.io/9fjdq/.

##### 2.1.3.1 Expected (dis)utility of disease and risk preferences

We modeled responses to disease vignettes using expected utility theory. Specifically, we computed the expected value of disease and associated treatment options to address the main question of decision-making under risk. Each illness presented an expected disutility of disease severity (EDS), computed from the expected utility theorem (Bernoulli, 1738/1954) as follows:

$\text{EDS}=P_{D}^{\;*\;}S_{D}.$

where p_D_ is the probability of contraction and S_D_ is disease severity. Each disease has a specific p_D_ (ranging from 0.05 to 0.50, see Tables 1, 2). S_D_ is a value on an ordinal scale ranging from 1 to 3, where 3 = death; 2 = severe lasting deficits and 1 = minor lasting deficits (the latter case was never used as a starting value, but only as the outcome of the sure option treatment).

##### 2.1.3.2 Linear models

We analyzed participants’ choices using a generalized linear mixed model with binomial distribution and Laplace approximation, to examine factors influencing decisions. First, we assessed the likelihood of making a costly choice (either certain or gamble) as opposed to inaction (Model 1a). Subsequently, we assessed, among costly choices, the likelihood of selecting a gamble over a sure option (Model 1b). In both models, we specified *EDS*, the sure option’s *Price* (0.1, 0.5 and 1 unit of monthly income), disease *Target* (*Self, Beloved* and *Stranger*), and the interaction thereof, as fixed factors. *EDS* and *Price* were specified as continuous predictors, whereas *Target* was treated as a categorical factor with three levels. Finally, we designed a linear mixed model to fit the treatment cost (i.e., the chosen treatment prices) as a function of the fixed factors *EDS, Target* and their interactions (Model 1c). In all three models, participant identity was specified as a random factor, with random intercept and slope for all within-subject predictors. In modeling the random components, we always chose the most complex random structure (slope of simple effects and high order interactions), except in cases of misconvergence, where a simpler structure was adopted (full details on the models implemented are provided in Supplementary Table A1). The analysis was performed using the *lmerTest* package of R (Kuznetsova et al., 2017).

### 2.2 Results

#### 2.2.1 Choice analysis

**Figure 1:**
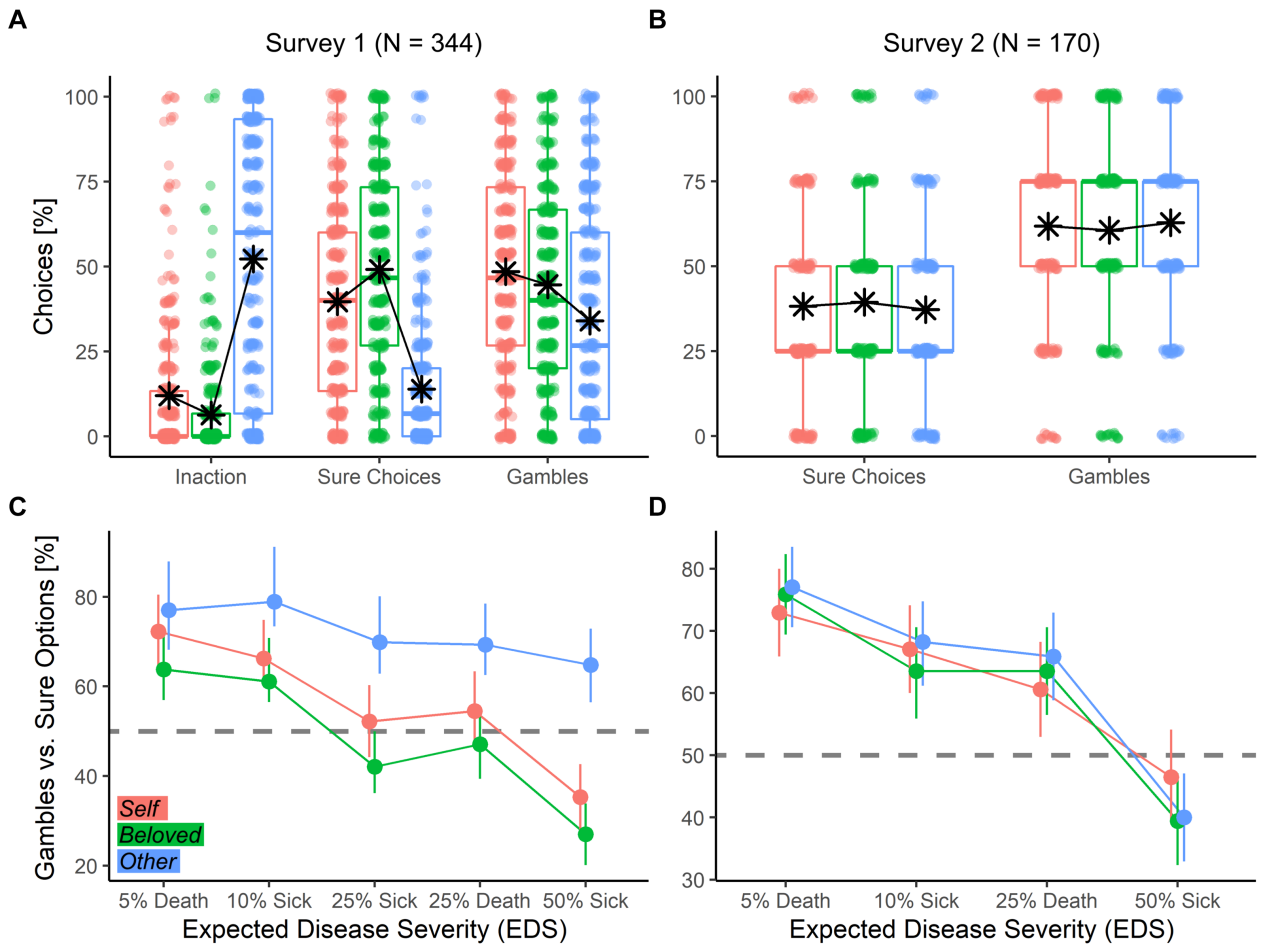
**(A,B)** Boxplots describing the percentage of each kind of choice across decision targets. For each boxplot, the horizontal line represents the median value of the distribution, the star represents the average, the box edges refer to the inter-quartile range, and the whiskers to the data range within 1.5 of the inter-quartile range. Individual data-points are also displayed as dots. **(C,D)** Line-graphs describing the relative percentage of Gambles vs. Sure Options across EDS and Target. Each condition is represented by the overall mean with bootstrap-based 95% confidence intervals. The horizontal dashed gray line shows the indifference point.

Overall, costly actions were significantly more frequent than inactions [76.53%, 26.67 interquartile range (IQR); test against 50%: *t*_(343)_ = 25.47, *p* < 0.001]. This effect was driven by *Self* and *Beloved* conditions, where costly actions were chosen most often (88.06%, IQR: 13.33 and 93.72%, IQR: 6.66, respectively). When choosing for a stranger (*Other* condition), costly options were selected less often, ~47.81% (IQR = 87.67; *t*_(343)_ = −1.03, *p* = 0.300; see Figure 1A). Among costly actions, participants preferred gambles (56.04%, IQR: 40.96; *t*_(341)_ = 4.25, *p* < 0.001). This effect was driven by *Self* and *Other* conditions, where gambles occurred 56.00% (IQR: 51.83) and 71.50% (IQR: 45.63) of the time, respectively. For the *Beloved*, gambles were chosen ~48.22% (IQR: 50.26; *t*_(340)_ = −1.10, *p* = 0.273) of the time.

**Figure 2:**
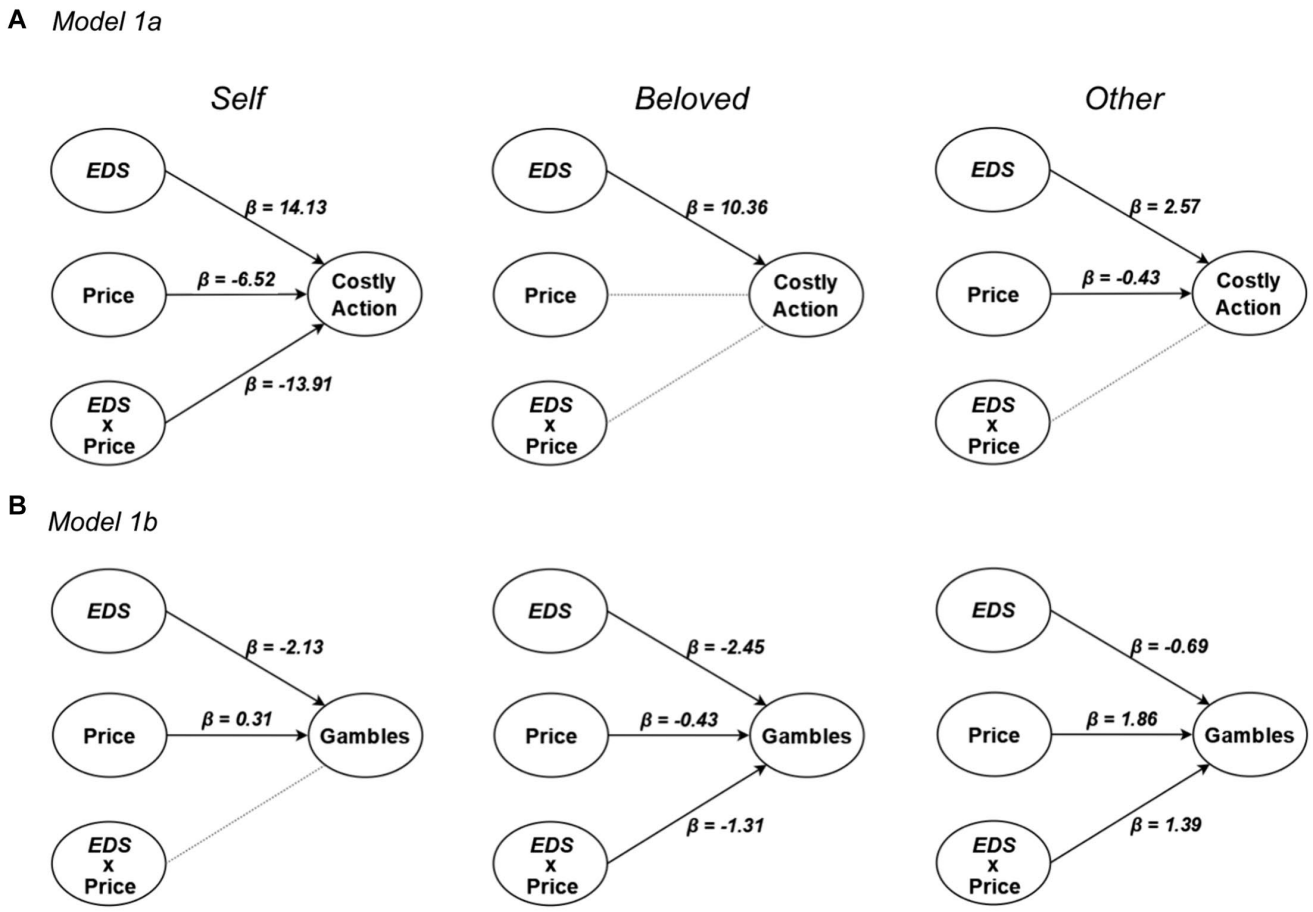
Results from simplified versions of **(A)** Model 1a and **(B)** Model 1b for *Self* (left), *Beloved* (middle) and *Stranger* (right) conditions. Significant effects are highlighted as full arrows associated with the corresponding *β* coefficient from a generalized linear mixed model with binomial distribution. Non-significant effects are displayed as dotted lines.

We extended our analysis in a generalized linear mixed model with a binomial distribution to assess factors affecting choice (Model 1a; Model 1b; Table 3). First, we found a positive effect of *EDS* on costly (Model 1a) and sure choices (Model 1b) (see Figure 1C; Table 3). We also found an effect of *Price*, with preferences for the cheaper option (Inaction in Model 1a; Gambles in Model 1b) as the sure option’s price increased; and an effect of *Target*, with fewer costly (Model 1a) and sure choices (Model 1b) made for the *Other* (see Figures 1A–C). In Model 1b, when participants chose a costly option, they gambled less for the *Beloved*. Finally, the three factors of interest interacted with one another, suggesting *EDS* and *Price* effects were conditioned on the Target. To further explore these interactions we repeated the previous models in each Target separately (Figure 2). Results confirm both *EDS* and *Price* influence self-regarding decisions in opposite directions. Whereas *EDS* promotes costly (Model 1a) and sure (Model 1b) choice selection, *Price* promotes inaction and gambles (Figure 2, left column). *Price* influences decisions for the *Beloved* less (Figure 2, middle column) while decisions for the *Other* are less influenced by *EDS* and more by *Price* (Figure 2, right column).

**Table 3:** Results of Survey 1.

Predictor	Model 1a		Model 1b		Model 1c	
	(Act. vs. inact.)		(Gamble vs. sure)		(Treatment cost)	
	*β*	*Z*	*β*	*Z*	*β*	*t*
Intercept	9.07	**10.30** ^***^	0.38	**2.59** ^**^	0.26	**−33.61** ^***^
EDS	12.97	**6.94** ^***^	−2.29	**−12.44** ^***^	0.13	**14.83** ^***^
Price	−0.73	**−2.35** ^*^	0.32	**2.31** ^*^	–	–
Target *Beloved*	−0.89	−0.80	−0.71	**−8.23** ^***^	0.06	**10.52** ^***^
Target *Stranger*	−8.94	**−9.99** ^***^	1.64	**9.16** ^***^	−0.15	**−19.98** ^***^
EDS*Price	−2.06	**−3.13** ^**^	0.25	0.75	–	–
EDS*Target *Beloved*	−3.90	−1.61	−0.22	−1.16	~0	−0.07
EDS*Target *Stranger*	−10.50	**−5.63** ^***^	1.43	**5.53** ^***^	−0.09	**−7.60** ^***^
Price*Target *Beloved*	1.07	**2.34** ^*^	−0.84	**−4.06** ^***^	–	–
Price*Target *Stranger*	0.39	1.16	1.69	**4.54** ^***^	–	–
EDS*Price*Target *Bel.*	3.21	**3.15** ^**^	−1.47	**−2.84** ^***^	–	–
EDS*Price*Target *Str.*	1.58	**2.19** ^*^	1.04	1.63	–	–

For each model, each fixed factor is described in terms of β coefficient and a statistical test (Z for binomial models, t for linear models) testing potential deviations from 0. Significant effects are highlighted in bold, with *** corresponding to *p* < 0.001, ** to *p* < 0.01, and * to *p* < 0.05.

#### 2.2.2 Chosen treatment price

**Figure 3:**
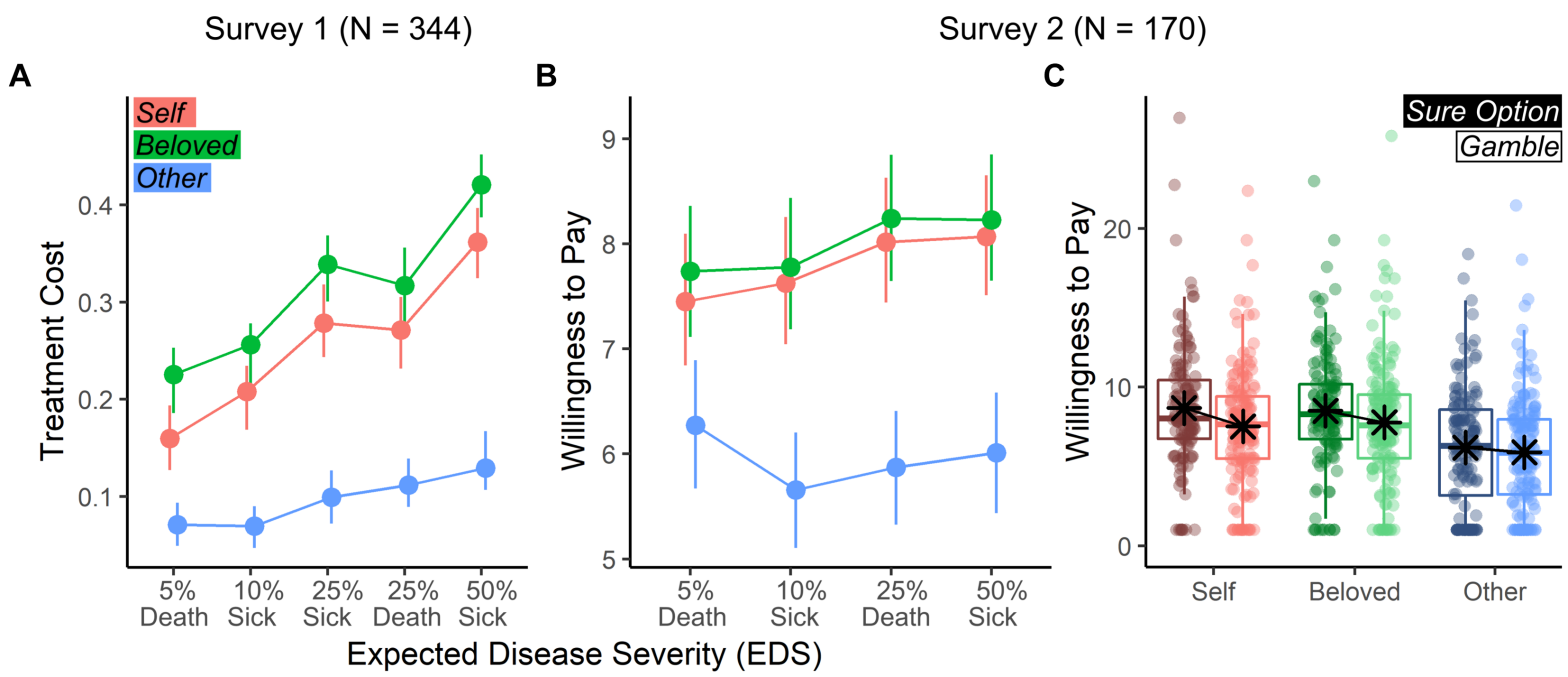
Treatment cost and willingness to pay. **(A)** Survey 1: Line-graphs describing the average cost (and bootstrap-based 95% confidence intervals) of the chosen treatment across EDS (horizontal axis) and Target (different color-coded lines). Costs are described as proportions of participants’ monthly salary. **(B)** Survey 2: Line-graphs describing the average willingness to pay of the chosen treatment described as log-transformed USD units. **(C)** Survey 2: Boxplots describing the willingness to pay across Target and previous choice. Values (in log-transformed USD) are displayed in different colors (to discriminate Targets) and luminance (to discriminate Choice).

In a follow-up model, the cost of the selected option was modeled as a dependent variable. Results (see Table 3 and Figure 3A) confirmed that participants spent more on the *Beloved*, but less on the *Stranger*. Furthermore, participants spent more for high *EDS* across targets, but this effect was less pronounced for the *Stranger*.

#### 2.2.3 Follow-up analyses

##### 2.2.3.1 Nuisance variables

We repeated all above analyses by accounting for *Sex, Age, Monthly Income* and COVID-19 information (e.g., log-transformed USD financial loss) as nuisance variables. Results confirm effects observed in the main analysis (Supplementary Table A2). We also found a significant effect of *Sex*, where males chose gambles less frequently (Model 1b) and accepted higher treatment prices (Model 1c).

##### 2.2.3.2 Alternative approach to EDS

All analyses reported were performed by modeling the predictor *EDS*, an adaptation of expected utility theory to health-based decision-making. In particular, *EDS* is defined as the product of the probability of contraction (p_D_) with disease severity (S_D_), where the latter is treated as a ratio-value although resulting from an ordinal predictor (e.g., 3 = death; 2 = severe lasting deficits and 1 = minor lasting deficits; Methods). As it could be argued that imposing linearity on S_D_ biases the analysis, we repeated the main analyses above, this time modeling the severity of disease as p_D_ + S_D_, as two independent predictors. Here, p_D_ was specified as a continuous predictor, and S_D_ as a categorical factor (in all diseases, initial S_D_ is either 2 or 3). Full results are displayed in Supplementary Table A3, and reveal that the effects originally attributed to EDS are now associated with p_D_. In some instances (albeit non-systematically) participants’ choices were also influenced by S_D_.

### 2.3 Discussion

This survey tested individual risky decision-making on other’s health using an expected disutility of disease framework. We found evidence supporting *Hypothesis 1* as individuals prefer gambles in the disease domain overall, with this preference decreasing linearly with expected severity of disease. This prediction was confirmed by our data (Figures 1A–C, rose data-points), similar to what found in the economic loss domain (Tversky and Kahneman, 1981) and pain management (Loued-Khenissi et al., 2022). More specifically, *EDS* and treatment price heavily influenced participants’ choices: whereas a disease of higher expected severity increased sure option selection, higher treatment cost increased gamble selection. Our prediction that individuals would be more risk averse for unknown others *(Hypothesis 2)* was not observed in our data, as participants selected sure options *less* frequently when acting for the *Stranger* (Figures 1A–C, blue data-points). Finally, *Hypothesis 3* predicted that choices for the *Beloved* would differ from those made for the *Stranger*, and be more similar to those observed for the *Self*. This was confirmed in our data, with increased risk aversion in the *Beloved* relative to the *Stranger* condition (Figures 1A–C, green data-points). In addition, participants exhibited the most risk-aversion when choosing for a loved one. The *Target* also affected the role played by *EDS* and *Price* on the decision, with choices for the *Beloved* more strongly influenced by *EDS*, while those for the *Other* were primarily price-based (Figure 2).

Finally, although our main analysis was framed on the estimation of a (dis)utility score for the disease (*EDS*), the results were not conditional to this choice. Similar effects were also observed when modeling the raw probability of disease contraction (p_D_).

## 3 Survey 2

In Survey 2, we explicitly differentiated risk preferences from cost concerns. For this purpose, we devised a modified version of Survey 1, where participants chose between sure and risky treatment options, and were subsequently asked to bid (in their own currency) on their chosen option. This measure of willingness-to-pay (WTP) has previously been used to valuate health interventions (Olsen and Smith, 2001). Importantly, removing the factor “price” made for a shorter survey of only 15 scenarios (5 diseases * 3 Targets). We further shortened the questionnaire to 12 scenarios (4 diseases * 3 Targets) to a survey that could be filled in ~15 min.

### 3.1 Methods

Unless otherwise stated, the set-up and analysis of Survey 2 were identical to those of Survey 1.

#### 3.1.1 Population

In Survey 2, respondents were not remunerated and were recruited through social media (Facebook, Twitter and LinkedIn) and survey swapping platforms.^3^ Within a time-window of 2 months (June–July 2020), 273 participants began the survey, and 175 completed the questionnaire. Five participants were excluded from analysis for providing implausible answers, leaving a sample of *n* = 170 for analysis. Cohort 2 was comparable to Cohort 1 for age (of both respondents and chosen *Beloved*), education and number of countries represented (Table 1). However, the cohorts differed on sex ratio and income (Survey 2 included more females and respondents reported a higher income).

#### 3.1.2 Procedure

Survey 2 included 12 scenarios with 4 diseases (Supplementary Table A1) affecting one of 3 possible targets (*Self, Beloved* and *Stranger*). Participants were asked to select between a sure option and a gamble, as in Survey 1. Respondents were not allowed to forgo action. Furthermore, no cost was associated with the options. Following choice, participants were asked to name a price [Willingness to Pay (WTP)], in their own currency for the chosen option. Respondents were explicitly told they could enter a value of 0 if they wished. Given its brevity, Survey 2 did not include any catch trials to assess attention.

#### 3.1.3 Data analysis

As in Survey 1, we first performed a choice analysis (Model 2a) testing whether *EDS, Target* or their interaction affected choice. Then we examined (*WTP*) as a dependent variable (Model 2b), with *EDS*, *Target*, previous *Choice* (Gamble vs. Sure Option) and their interaction as predictors of interest (Supplementary Table A1). The WTPs were converted from participants’ local currency to USD (based on the official exchange rate on the day of their response), and log-transformed to account for the large range in responses.

Each model was associated with a power analysis to test whether the current design at a given sample size would be sufficiently powered to replicate findings from Survey 1. Estimates of fixed factors coefficients and random-effect terms for Model 2a were obtained by re-analyzing Models 1b from Survey 1 without the factor “price.” As for Model 2b, we took the coefficients/terms obtained from the analysis of treatment price (Model 1c), although this model provides only partial information as no factor “choice” was specified in Survey 1. For each model, and each main/interaction effect, we ran 1,000 Monte-Carlo simulations aimed at replicating the same fixed factors coefficients and random-effect terms observed in Survey 1 on the design and sample size from Survey 2. Power was then estimated from the frequency of significant effects from the simulated data, as implemented in the *simr* package of R (Green and MacLeod, 2016). This analysis showed that the design and sample size were sufficiently sensitive to replicate the effects of *EDS* and *Target* observed in Model 1b from Survey 1 with a probability of at least 0.88.

### 3.2 Results

#### 3.2.1 Choice analysis

As in Survey 1, participants preferred gambles over sure options (62.72%, IQR: 25.00; test against 50%: *t*_(169)_ = 6.18, *p* < 0.001), an effect observed in all three targets with comparable percentages. We further inspected choice preferences through a generalized linear mixed model, under binomial distribution (Model 2a). Results confirmed the same effect of *EDS* observed in Survey 1 (Table 4), where gambles decreased with increasing *EDS* (Figure 1D). We found no effect of *Target.* Overall, the analysis of Survey 2 revealed that when risk preferences are dissociated from cost, *Target* effects disappear.

**Table 4:** Results of Survey 2.

Predictor	Model 2a		Model 2b	
	(Gamble vs. sure)		(Willingness to pay)	
	*β*	*Z*	*β*	*t*
Intercept	0.71	**4.57** ^***^	7.99	**25.67** ^***^
EDS	−1.57	**−4.75** ^***^	0.40	1.51
Choice	–	–	−0.36	**−2.63** ^**^
Target *Beloved*	0.09	0.51	0.11	0.81
Target *Stranger*	0.08	0.50	−2.00	**−7.40** ^***^
EDS*Choice	–	–	0.33	0.91
EDS*Target *Beloved*	−0.69	−1.48	0.18	0.50
EDS*Target *Stranger*	−0.48	−1.11	−0.67	−1.67
Choice*Target *Beloved*	–	–	0.15	0.85
Choice*Target *Stranger*	–	–	0.31	1.52
EDS*Choice*Target *Bel.*	–	–	−0.27	−0.57
EDS*Choice*Target *Str.*	–	–	0.04	0.08

For each model, each fixed effect is described in terms of β coefficient and a statistical test testing potential deviations from 0. Significant effects are highlighted in bold, with *** corresponding to *p* < 0.001, ** to *p* < 0.01, * to *p* < 0.05.

#### 3.2.2 Willingness-to-pay

In contrast to risk preferences, *Target* influences *WTP*, with participants bidding less on the *Stranger* (Table 4; Figure 3B). Additionally, we found an effect of previous *Choice*, with participants bidding more on sure options than gambles (Figure 3C). However, only 24.71% of trials listed a 0 bid for the *Stranger*, indicating a persistence of altruism and prosocial motivation.

#### 3.2.3 Follow-up analyses

##### 3.2.3.1 Nuisance variables

We repeated analyses including Sex, Age, Monthly Income and COVID-19 information as nuisance variables. Results confirmed all effects observed in the main analysis (Supplementary Table A4), with the exception of the Choice effect from Model 2c (*β* = −0.31, *t*_(627.62)_ = −1.70, *p* = 0.090). When analyzing the effect of the nuisance variables, we found a significant positive effect of COVID-19 financial loss on WTP (Supplementary Figure A1), suggesting that participants who sustained a higher financial loss due to the pandemic were willing to pay more for others. This was observed by specifying both monthly income and financial loss in the same model, indicating this effect was not confounded with personal wealth.

##### 3.2.3.2 Alternative approach to EDS

As in Survey 1, we repeated the analyses of the main models by replacing EDS with p_D_ + S_D_, as two independent predictors. Full results are displayed in Supplementary Table A5, and reveal that all effects originally attributed to EDS are now associated with p_D_. No effect was associated with S_D_.

### 3.3 Discussion

Survey 2 confirms both *Hypothesis 1* and the first result from Survey 1 in that, in the *Self* condition, individuals display risk-seeking behavior in the disease domain, as highlighted by the preference toward gambles vs. sure options. Furthermore, this preference for gambles decreases linearly with *EDS*. However, when differentiating risk preferences from cost concerns, Survey 2 results show no *Target* difference, going against the predictions of *Hypotheses 2 and 3* (Figures 1B–D). Instead, target differences were observed only in the analysis of WTP, with participants bidding less to treat the *Stranger* (Figure 3B). This result disambiguates an open issue from Survey 1, suggesting target differences in other-regarding decision-making under risk are conditioned on cost considerations, and not risk preferences. Finally, while respondents preferred gambles overall, WTP analyses reveal a higher monetary value placed on sure options (Figure 3C).

## 4 General discussion

The goal of this study was to probe decision-making under risk in health interventions for self and others in the context of the COVID-19 pandemic’s early days. The study was specifically aimed at gaining a cross-sectional description of how a lay individual selects costly actions on behalf of another person’s health, under risk. As authorities called on the public to act for others’ sake during the pandemic, the burden of uncertain decision-making was thrust onto individual shoulders.

### 4.1 Individuals are risk-seeking for health treatments

Across two surveys, we confirm the first hypothesis of this study, according to which individuals prefer to gamble to prevent disease for themselves. This effect is in line with studies on different kinds of negative rewards, ranging from monetary loss (Tversky and Kahneman, 1981), to pain prevention (Loued-Khenissi et al., 2022). It is possible that the framing of decision outcomes in the present study as negative (getting sick) could have influenced the results toward a pattern similar to that of monetary losses (Tversky and Kahneman, 1981). In this perspective, an alternative framing with a positive outcome (being healed) could in principle lead to diverging results. However, surveys targeted the general population that, while in good health, was confronted with the risk of contracting COVID-19 (as the survey was conducted in the early days of pandemic, almost no-one of our participants contracted SARS-Cov-2 virus, Table 1). Casting the retention of improved health as a positive outcome in this population may have stretched responders’ credulity. This may differ in patient populations, where treatments for different disease scenarios could be realistically framed as a positive shift from their present condition.

Critically however, though participants displayed an overall preference for gambles, they became progressively more risk-averse with higher expected disease severity. This finding is consistent with previous research in the domain of economic decision-making (Tversky and Kahneman, 1981) and pain management (Loued-Khenissi et al., 2022). In addition to confirming our predictions, these results put forward the effectiveness of expected utility models in explaining health decision-making (Cohen, 1996). Although several studies criticize such an approach for health decisions (Abellan-Perpiñan et al., 2009; Dolan and Kahneman, 2008), we argue that expected utility is a useful tool for modeling individual behavior, in line with what is known on life quality (Attema et al., 2016), pain management (Loued-Khenissi et al., 2022), as well as brain activity (Knutson et al., 2005; Loued-Khenissi et al., 2020; Schultz, 2016). Importantly, our results are not idiosyncratic to the theoretical framework adopted in our study, as similar effects were obtained when replacing *expected disease severity* with the raw probability of disease contraction (p_D_). Hence, absent any explicit requests to compute probabilistic outcomes, individuals in our study appear to choose according to those quantities nonetheless. In this perspective, concerns over individuals’ difficulty in understanding probabilities, particularly in the context of the pandemic (Aguilar and Castaneda, 2021; Muñiz-Rodríguez et al., 2020) are not supported, and therefore authorities should consider informing the public in accurate, probabilistic terms (Kahlenberg et al., 2023).

Although individuals selected gambles more often, they simultaneously assigned a higher monetary value to sure treatment options (Figure 3C). This effect is known as *preference reversal* (Safra et al., 1990; Seidl, 2002), prevalent in economic frameworks but also observed in the domain of pain management (Loued-Khenissi et al., 2022). Although the cause of preference reversals is still debated, scholars attribute it to the sequential nature of many paradigms that distort pricing estimates, or to a general tendency to overprice options with high probability and low benefit at the expense of options with low probability and high benefit (see Seidl, 2002, for a review). Both these explanations fit the case of Survey 2, further stressing how choices in the context of disease prevention dovetail with predictions based on theories of economic decision-making.

### 4.2 Target differences are explained by cost considerations

In both surveys, participants’ behavior differed as a function of disease target, especially for strangers. In Survey 1, respondents selected gambles more often for strangers than for themselves or their loved ones (Figure 1C). *Prima facie*, these results suggest a stronger risk-seeking stance for other-regarding decisions. However, Survey 1 results may also reflect the fact that the price associated with gambles in the survey was (1) stable across trials, and (2) cheaper or equal to that of sure options. Participants’ behavior toward strangers was also characterized by a high amount of inactions (Figure 1A) where participants refrained from choosing to avoid incurring personal cost. In Survey 2, where choices were embedded in a cost-free context, individuals risk preference for others was the same as that observed for self-regarding behavior. Individuals diverged in action for themselves and unknown others only with respect to willingness-to-pay. We therefore propose that, when cost is not a factor in decision-making, risk biases do not have a differential impact on self and others. However, when cost *is* a factor, decisions differ between targets by pushing agents toward a value-based heuristic, where cheaper options are preferred for strangers.

It is unclear why, in the present study, risk preferences remain the same across the self-other boundary, something that contrasts with prior research that have found a dissociation (Atanasov, 2015; Loued-Khenissi et al., 2022; Polman and Wu, 2020). Two considerations emerge from this finding. First, results provide evidence that individuals deploy a simple self-referential strategy when computing uncertainty for others (at least in contexts that are cost-free and anonymous), possibly to minimize cognitive demand (Tomova et al., 2020). Second, although previous meta-analyses report overall self-other differences in risk preferences these effects are extremely variable between studies, pointing to a wide range of moderators that influence participants’ choices (Atanasov, 2015; Polman and Wu, 2020). Among these are the framing of the context (e.g., involving positive vs. negative outcomes, financial vs. medical decisions) and, most critically, the identity of the target (adult, child, patient) and his/her personal relationship with the deciding agent (family member, colleague, stranger) (Atanasov, 2015; Polman and Wu, 2020). It is possible that any of these moderating factors (or the combination thereof) might have influenced our results with respect to prior literature.

Instead, the fact that target differences (across the self-stranger boundary) emerge only when monetary cost becomes a relevant parameter to an agent is consistent with current theoretical accounts. For instance, Lockwood et al. (2017) found that effortful (costly) pro-social choices triggered apathy, thereby suggesting that one main discriminant between self vs. others decision-making lies in resource mobilization. Most importantly, these results are also in line with predictions from evolutionary theory on kinship and indirect fitness (Kay et al., 2019), according to which costly behavior might be evolutionarily advantageous only when benefiting close ones, at the expense of strangers. It should be stressed, however, that strangers still received treatments that were more expensive than the cheapest option: in Survey 1, costly choices were chosen ~48% of the time, while in Survey 2 participants consistently made bids higher than 0. Based on these results, individual behavior for strangers does not mirror self-regarding behavior—but neither does it reflect purely or even mostly selfish motivations. On the contrary, participants were willing to incur costly prosocial behavior even when kinship motives are absent, as for the *stranger* condition in our study. These altruistic tendencies have previously been found in several researches from behavioral economics (Fehr and Fischbacher, 2004; Fehr and Schmidt, 2006; Frey and Meier, 2004) and more generally in the field (Sisco and Weber, 2019). They offer a valuable insight into the boundaries one can expect from individuals for the sake of others’ wellbeing, especially in scenarios with high uncertainty, rather than relying on heuristics that may backfire (Bonaccorsi et al., 2020; Fink et al., 2022; Wood et al., 2022) and compromise political trust (Jørgensen et al., 2022).

It could be argued that these donations are the result of a so-called *experimenter effect*, where participants are motivated by reputation concerns (Hoffman et al., 1996). The experimenter effect has been observed in tasks such as the dictator game, where anonymity between the participant and the experimenters decreased the amount of free donations (Hoffman et al., 1996). However, as our study guaranteed full anonymity, we believe that the risk of such confounds are negligible. Furthermore, participants’ WTP in Survey 2 was positively influenced by real confinement-related financial loss (while controlling for personal wealth). This hints toward a genuine pro-social disposition held by respondents to provide for others’ wellbeing, including strangers. These results are in line with previous environmental research measuring willingness-to-pay for options that benefit members of a future generation. Even in those scenarios where delay-discounting can dampen pro-social motivations, people exhibit a positive attitude toward others’ wellbeing (Graham et al., 2019).

### 4.3 Choices for loved ones resemble those made for the self

Our third hypothesis predicted that individuals’ behavior toward a stranger would differ from that made for a loved one, in that the latter would trigger decisions more similar to those made for the self. When considering risk preferences alone, we found no target differences, thus providing no support for our prediction. However, when taking into account cost-considerations, we find evidence supporting this hypothesis. Whereas participants chose cheaper options for a stranger, the chosen cost for treating a loved one was either higher than (Survey 1) or comparable to (Survey 2) that chosen for the self. This effect is reminiscent of what is found in the literature on pain decisions, where individuals’ behavior and susceptibility to risk differs strongly between an unknown other and a loved one, with the latter resembling those made for the self (Loued-Khenissi et al., 2022). This result also conforms to the empathy model from social neuroscience literature, where individuals treat others’ suffering as their own by triggering the same neural processes that mediate direct pain experience (Bernhardt and Singer, 2012; Corradi-Dell’Acqua et al., 2011, 2016, 2023). This model acknowledges a strong role played by social proximity, with less pronounced empathic responses for those deemed distant from the self (Cheng et al., 2010; Hein et al., 2010; Xu et al., 2009).

Although similar to one another, responses associated with the self and a loved one were not identical. In particular, in Survey 1, choices made for a loved one differed in several ways from the self-condition: they were more risk averse, less influenced by price and more by *EDS* (or p_D_, depending on the analysis), and assigned a higher monetary value. These results show that social proximity shifts one’s behavior from wellbeing-oriented (for close others) to price-oriented (for strangers; see Figure 2B). These effects were not observed in Survey 2, though a power analysis established that the sample collected was adequate to reproduce *Target* differences from Survey 1. It is possible that these effects manifest themselves only in complex settings where price and *EDS* are integrated together. However, it should also be mentioned that the power analysis tested effects of *Target* as a whole: i.e., across all three levels. It is therefore possible that the results were influenced by the strong modulations of the *Stranger* condition, and that a more sensitive cohort would have been necessary to replicate subtle *Self* vs. *Beloved* differences.

### 4.4 Limitations of the study and future implications

This study has three limitations that need to acknowledged. First, Survey 2 different slightly from Survey 1 in that: it was shorter, it was targeted to unpaid volunteers recruited outside Prolific.co platform, and no catch trials were implemented to monitor participants’ attentional level. It is in principle possible that some participant in Survey 2 lost focus during the task despite its brevity. More critically, the two surveys might have probed slightly different populations, by attracting individuals with different financial status (Table 1). Second, recruitment for unpaid volunteers for Survey 2 was more time consuming. Given the rapid pandemic progression, it is possible that perception of risk for people’s health changed across time. Hence, a much delayed recruitment time would have exposed us to the risk of probing a different situational cohort with respect to Survey 1. We minimized such possibility by interrupting data collection following 2 months so that participants from Survey 1 (tested on May 2020) and those from Survey 2 (June–July) were tested in close proximity. The drawback of this choice was that the sample size was imbalanced between the surveys, with Survey 2 being limited to 170 participants. However, rigorous simulation-based power analysis insured that such sample was sufficiently sensitive to replicate the effects of interest from Survey 1. Third, studies on economic decision making and prosocial behavior often report big inter-individual differences, explainable in terms of personality or empathic traits (Thielmann et al., 2020), prosocial beliefs (Carlson and Zaki, 2022) as well as COVID-19 information (disease contraction, regional death rate, etc.; Fang et al., 2022). Unfortunately, personality/social traits were not collected in this study, preventing us to assess these effects also in our dataset. We did collect information about individual COVID-19 experience (see methods for the measures collected), but these measure were either unsuitable for statistical analyses (positive cases of SARS-Cov-2 virus were negligible; Table 1) or did not reveal reliable influence on choice behavior.

Keeping these considerations aside, our study provides novel and replicable evidence on how people make decisions about one’s own other people’s health under risk. In particular, we found that individuals act for their own health as is observed in the monetary loss domain, by displaying an overall risk-seeking stance that progressively declines as the expected value of a negative event increases. This effect did not differ statistically when choices under risk were made for others, at least when cost considerations were put aside. However, distinctions between decision targets emerged when choices were conditioned on monetary cost, with participants preferring cheaper treatment options for unknown others, but not for loved ones.

The COVID-19 pandemic imposed the burden of costly decision-making under risk for others’ health on ordinary people. Most of the restrictive measures implemented across the globe had negative economical, but also psychological consequences on the people involved (Kunzler et al., 2021; Lima et al., 2020; Nochaiwong et al., 2021; Santomauro et al., 2021; Wu et al., 2021). These restrictions negatively impacted sensitivity to others’ suffering, and empathy traits (Antico and Corradi-Dell’Acqua, 2023; Cao et al., 2022), thus raising the question on what cost lay individuals were willing to incur for the sake of a stranger, and this under uncertainty. In this perspective, our study provides a descriptive model of individual risky decision-making in health-contexts; and inform on the limits of what can be asked of an individual in service to a stranger. Furthermore, as global society faces looming events such as climate change (Hornsey et al., 2022) or migration (Denniston, 2021) that demand individual participation for their mitigation, it is crucial to gain an understanding of factors influencing other-regarding decision-making under uncertainty. As respondents showed a readiness to incur cost for the sake of strangers, authorities can assume a general goodwill and willingness to help others (albeit at a lower rate of cost to that observed in self-regarding decisions or decisions made for loved ones), underscoring our tendency toward pro-sociality.

## Data Availability

The datasets presented in this study can be found in online repositories. The names of the repository/repositories and accession number(s) can be found at: Open Science Framework: https://osf.io/9fjdq/.

## Footnotes

^1^ https://www.prolific.co/

^2^ https://cran.r-project.org/

^3^ https://www.surveycircle.com/
