# Supplementary material for: Gambling on others’ health: risky pro-social decision-making in the era of COVID-19
Source: Front Psychol. 2024 Sep 19;15:1370778. doi: 10.3389/fpsyg.2024.1370778 (PMC11450482; doi:10.3389/fpsyg.2024.1370778)

Appendixes

Table A1

Details of each linear model implemented in the Study. Models 1c and 2b were Linear Mixed Model, and effect significance was calculated using the Satterthwaite approximation of the degrees of freedom. Instead, for models 1a, 1b and 2a, we employed a Generalized Linear Mixed Model with a binomial distribution and Laplace approximation.

**Model**	**Dependent Variable**	**Detailed model** (*lmer syntax)*
1a†	Choice *(Costly Action vs. Inaction)*	*Choice ~ EDS*Target*Price + (EDS*Target+Price| Subjects)*
1b	Choice *(Gamble vs. Sure option)*	*Choice ~ EDS*Target*Price + (EDS*Target*Price| Subjects)*
1c	Treat. Cost *(Chosen Price)*	*Treat. Cost ~ EDS*Target + (EDS*Target | Subjects)*
2a	Choice (*Gamble vs. Sure option*)	*Choice ~ EDS*Target + (EDS*Target | Subjects)*
2b†	Willingness to Pay (WTP)	*WTP ~ EDS*Target*Choice + (EDS*Target + EDS*Choice | Subjects)†*

†As the model with the most complex random structure did not reach convergence, a model with a simpler structure was instead selected.

Table A2

Follow-up analysis of Survey 1: control for nuisance variables. Results from the same models described in Table 7 with the inclusion of four nuisance variables.

Predictor	***Model 1a*** *(Act. vs. Inact.)*		***Model 1b*** *(Gamble vs. Sure)*		***Model 1c*** *(Treatment Cost)*	
	β	*Z*	*β*	*Z*	*β*	*t*
Intercept	5.66	**12.30*****	0.82	**3.86****	0.23	**-21.46*****
EDS	5.33	**11.60*****	-2.09	**-13.56*****	0.14	**13.65*****
Price	-1.08	**-3.08****	0.24	1.76	--	--
Target *Beloved*	0.60	1.36	-0.66	**-7.59*****	0.05	**8.81*****
Target *Stranger*	-5.82	**-13.83*****	1.35	**7.91*****	-0.16	**-17.58*****
EDS*Price	-2.57	**-3.43*****	0.18	0.63	--	--
EDS*Target *Beloved*	0.25	0.44	-0.13	-0.87	0.01	0.57
EDS*Target *Stranger*	-2.69	**-6.39*****	1.07	**5.59*****	-0.09	**-7.11*****
Price*Target *Beloved*	1.45	**2.87****	-0.45	**-2.52***	--	--
Price*Target *Stranger*	0.73	1.94	0.82	**3.43*****	--	--
EDS*Price*Target *Bel.*	4.46	**3.67*****	-0.47	-1.20	--	--
EDS*Price*Target *Str.*	2.03	**2.48***	1.02	**2.21***	--	--
Gender	0.29	0.66	-0.60	**-2.29***	0.04	**3.31****
Age	-0.01	-0.64	-0.02	-1.26	~0	1.17
Monthly Income	0.36	1.25	-0.11	-0.61	0.01	1.69
Covid-19 Loss	0.05	0.94	0.01	0.43	~0	0.41

Table A3

Follow-up analysis of Survey 1: alternative approach to *EDS*. For Models 1a, 1b and 1d, *EDS* is replaced by two separate orthogonal predictors: disease probability (p_D) and severity (S_D) as continuous and categorical factors respectively.

Predictor	***Model 1a*** (*Act. vs. Inact.*)		***Model 1b*** (*Gamble vs. Sure*)		***Model 1d*** (*Treatment Cost*)	
	β	*Z*	*β*	*Z*	*β*	*t*
Intercept	6.21	**13.43*****	0.84	**6.03*****	0.24	**27.02*****
p_D	0.18	**10.62*****	-0.08	**-10.91*****	0.01	**11.51*****
S_D	-0.80	**-3.55*****	0.37	**3.25****	-0.15	-1.85
Price	-0.41	-1.30	0.23	1.39	--	--
Target *Beloved*	-0.13	0.28	-0.64	**-6.36*****	0.05	**6.84*****
Target *Stranger*	-7.02	**-14.30*****	1.13	**6.55*****	-0.15	**-16.11*****
p_D*Price	-0.05	-1.88	0.08	0.32	--	--
S_D*Price	0.37	0.88	0.06	0.22	--	--
p_D*Target *Beloved*	-0.04	**-2.25*****	~0	0.19	~0	-0.57
p_D*Target *Stranger*	-0.08	**-4.60*****	0.01	1.11	-0.003	**-5.19*****
S_D*Target *Beloved*	0.42	1.56	-0.15	-1.15	~0	0.04
S_D*Target *Stranger*	1.24	**5.11*****	-0.17	-1.00	0.03	**2.38***
Price*Target *Beloved*	0.40	0.82	-0.56	**-2.46***	--	--
Price*Target *Stranger*	0.14	0.37	0.73	**2.28***	--	--
p_D*Price*Target *Bel.*	0.06	1.55	0.02	0.77	--	--
p_D*Price*Target *Str.*	0.01	0.48	~0	-0.06	--	--
S_D*Price*Target *Bel.*	-0.55	-0.85	0.15	0.45	--	--
S_D*Price*Target *Str.*	-0.34	-0.64	-0.07	-0.15	--	--

Table A4

Follow-up analysis of Survey 2: control for nuisance variables. Results from the same models described in Table 7 with the inclusion of four nuisance variables.

Predictor	***Model 2a*** (*Gamble vs. Sure*)		***Model 2b*** (*Willingness to Pay*)	
	β	*Z*	*β*	*t*
Intercept	0.61	**2.84*****	8.35	**17.81*****
EDS	-1.76	**-4.09*****	0.60	1.73
Choice	--	--	-0.31	-1.70
Target *Beloved*	-0.03	-0.14	0.19	1.06
Target *Stranger*	0.03	0.14	-1.95	**-5.32*****
EDS*Choice	--	--	0.22	0.43
EDS*Target *Beloved*	-0.52	-0.88	0.14	0.31
EDS*Target *Stranger*	-0.61	-1.11	-0.96	-1.77
Choice*Target *Beloved*	--	--	0.20	0.86
Choice*Target *Stranger*	--	--	0.19	0.72
EDS*Choice*Target *Bel.*	--	--	-0.02	-0.03
EDS*Choice*Target *Str.*	--	--	0.17	0.25
Gender	0.44	1.52	-1.01	-1.56
Age	~0	0.05	-0.01	-0.21
Monthly Income	0.06	0.41	0.63	1.76
Covid-19 Loss	-0.01	-0.16	0.24	**3.06****

Table A5

Follow-up analysis of Survey 2: alternative approach to *EDS*. Models 2a and 2c, *EDS* is replaced by two separate orthogonal predictors: disease probability (p_D) and severity (S_D) as continuous and categorical factors respectively.

Predictor	***Model 2a*** *(Gamble vs. Sure)*		***Model 2c*** *(Willingness to Pay)*	
	β	*Z*	*β*	*t*
Intercept	0.57	**3.02**[**]	7.88	**24.15**[***]
p_D	-0.03	**-4.14**[***]	0.01	1.51
S_D	0.38	1.49	0.27	1.24
Choice	--	--	-0.23	-1.21
Target *Beloved*	-0.24	-1.08	0.09	0.47
Target *Stranger*	-0.01	-0.06	-1.99	**-6.50**[***]
p_D*Choice	--	--	~0	0.89
S_D*Choice	--	--	0.26	-0.94
p_D*Target *Beloved*	~0	-0.95	~0	0.44
p_D*Target *Stranger*	~0	-0.86	-0.10	-1.17
S_D*Target *Beloved*	0.47	1.43	0.08	0.28
S_D*Target *Stranger*	0.16	0.51	-0.02	-0.08
Choice*Target *Beloved*	--	--	0.11	0.42
Choice*Target *Stranger*	--	--	0.12	0.44
p_D*Choice*Target *Bel.*	--	--	~0	-0.39
p_D*Choice*Target *Str.*	--	--	~0	-0.16
S_D*Choice*Target *Bel.*	--	--	0.03	0.08
S_D*Choice*Target *Str.*	--	--	~0	0.08

Figure A1

Survey 2: Scatter Plot with linear regression lines (and 95% confidence interval area) describing the relationship between Willingness to Pay against Covid-19 Financial Loss (both in log-transformed USD units). The Spearman's correlation coefficient are also displayed with significance highlighted as follows: "***" $p < 0.001$.

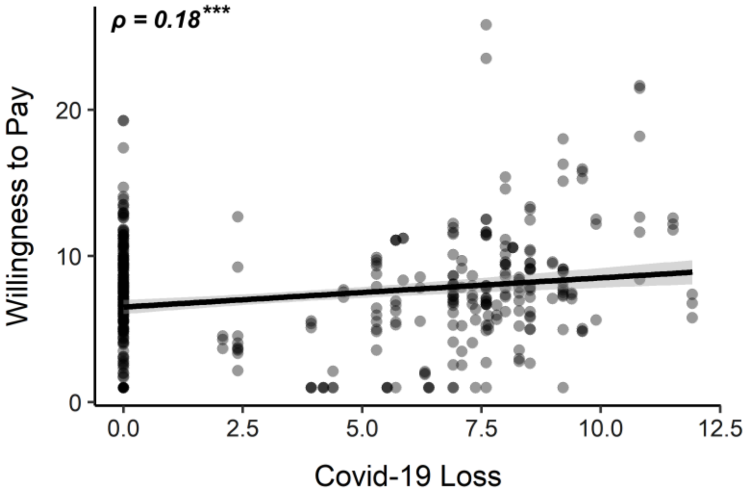

Supplement: Supplementary file 1 [file Presentation_1.pdf]
